# A dynamic novel approach for bid/no-bid decision-making

**DOI:** 10.1186/s40064-016-3230-1

**Published:** 2016-09-15

**Authors:** Huawang Shi, Hang Yin, Lianyu Wei

**Affiliations:** 1 School of Civil Engineering, Hebei University of Engineering, Handan, 056038 Hebei China; 2 School of Civil Engineering, Hebei University of Technology, Tianjin, 300401 China

**Keywords:** Rough sets, GRNN neural network, Niche particle swarm optimization, Bid/no-bid decision

## Abstract

The process of bid/no-bid decision-making is su bjected to uncertainty and influence of complex criteria. This paper proposed an application of the integration of rough sets (RS) and improved general regression neural network (GRNN) based on niche particle swarm optimization (NPSO) algorithm for tendering decision making. The decision table of RS and the attribution reduction was processed by MIBARK algorithm to simply the samples of GRNN. In order to improve the general regression neural network (GRNN) network performance, the niche particle swarm optimization (NPSO) was used to optimize the spread parameter *σ* of GRNN neural network, then a novel Bid/no-bid decision model was established based on RS and NPSO-GRNN neural network algorithm. The applicability of the proposed model was tested using real cases in Beijing. The results indicate that NPSO-GRNN algorithm has an advantage such as in prediction accuracy and generalization ability. The proposed decision support system approach is useful to help manager to make better Bid/no-bid decisions in uncertain construction markets, so they can take steps to prevent bid distress.

## Background

When a letter of call for bid has been received, construction manager must decide to submit a bid or not. For any construction firms, being able to make right bidding decision is very important. Biding or not is a very important activity for a contractor (Lin and Chen 2004; Mahdi and Alreshaid 2005; Irtishad 1990).

To aid managers in bid/no bid decisions making, many decision methods for bidding have been proposed to assist the construction managers making better decision-making in an uncertain biding environment. Many scholars have proposed techniques for bid decision-making. However, traditional models for bid decision-making tend to utilize quantitative tools, just as economic models, mathematical programming, etc. which managers in both theoretical and practical did not show interest in such models (Irtishad 1990). The complexity of the problem is so overwhelming that even the very experienced contracting managers feel that the bid/no-bid decisions should have a better technique tool for archiving. El-Mashaleh (2013) concluded key bidding variables that are considered by contractors when evaluating bids. Senior managers of contracting industry were interviewed to identify variables that affect biding and data envelopment analysis (DEA) developed to use in the tender decision. Boussabaine and Lewis (2003), proposed a novel tender decision method utility the artificial neural network (ANN) technique. A back-propagation ANN consisting of an input layer with 18 input nodes, two hidden layers and output layer with one node was developed. Chou et al. (2015) proposed an method for estimating project award prices utilizing artificial intelligence (AI)-based bid/no bid technical as well as an auxiliary tool that contract managers can use to make bid/no bid decision-making. This research optimizing AI models that predict bid award amounts for bridge projects. Chou et al. (2013) developed a new bid/no bid decision-making strategy to support the decision-making that is based on a combined framework of the Fuzzy Analytical Hierarchy Process (FAHP) and regression-based simulation. In a word, new methods of artificial intelligence(AI) have been widely used in tender decision with the increasing development of science and computer technology (Deng et al. 2015; Jiang et al. 2015). Among them, the BP artificial neural networks(ANN) algorithm has been extensive used, but the practical application of the algorithm has certain limitations due to it is easily trapped in local minima and the poor convergence performance. In view of some defects existing in the traditional bid/no bid forecast methods and the problem of insufficient predicted sample amount of historical data, this paper developed a novel approach integrating rough sets with GRNN neural network based on NPSO algorithm to bid/no-bid decisions. It can not only overcoming the defects that the network is easy to fall into local minimum, poor convergence and etc., but also improve and optimize the generalization capability and performance of the network through NPSO- GRNN neural network algorithm.

The organize of this paper is structured as: Introduction of bid/no bid decisions making are presented in section “Background”. The basics of NPSO and GRNN methodology are introduced. The framework and key algorithms are proposed, and the flowchart of proposed NPSO- GRNN approach is designed in section “Methods”. Data analysis, model implementation and some comparisons are put forward to demonstrate the developed approach in section “Data analysis and model implementation”. Results and discussion are listed in section “Results and discussion”. Our conclusions and expectations are summarized in section “Conclusions”.

## Methods

### GRNN model

**Fig. 1:**
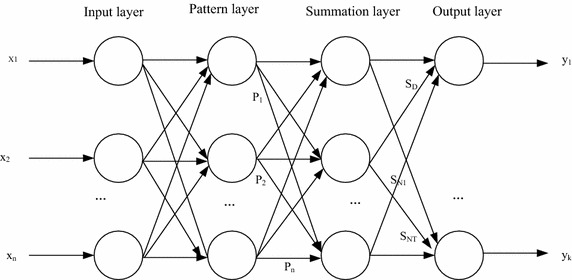
GRNN network structure

General regression neural network was developed by the Donald F.Specht in 1991, which is a radial basis function neural network (Chongzhen and Jingguo 2009; Chen et al. 2012). As shown in Fig. 1. GRNN network structure is composed of four layers.

The four-layer structure of GRNN network is as follow: the input layer, the pattern layer, the summation layer and the output layer. Let the input vector is: $X = \left[ {x_{1} ,x_{2} , \ldots x_{n} } \right]^{T}$, output vector is: $Y = \left[ {y_{1} ,y_{2} , \ldots y_{k} } \right]^{T}$.

The input neurons nodes are equal to input vector dimension in the learning sample, each node which is a simple distribution unit directly takes the input vectors into the pattern layer.

The pattern layer neuron conversion formula is:

1 $${\text{P}}_i = \exp \left[ { - \frac{{(X - X_i)^{T} (X - X_{\text{i}})}}{{2\sigma^{2} }}} \right]\quad {\text{i}} = 1,2, \ldots ,{\text{n}}$$

X is input variable for the network, and $X_i$ = the *i*th neuron correlated learning samples, *σ* represents spread parameter.

The summation layer processes with two summation method. The first category is calculated as:

2 $$\sum\limits_{i = 1}^{n} {\exp \left[ { - \frac{{(X - X_i)_{{}}^{T} (X - X_i)}}{{2\sigma_{{}}^{2} }}} \right]}$$

Equation (2) is exponent summation of all output of nodes in the pattern layer, the connection weights for each model layer and neurons is 1, the conversion formula is $SD = \sum\nolimits_{i = 1}^{n} {P_{i} }$.

The second category is calculated as

3 $$\sum\limits_{i = 1}^{n} {Y_i\exp \left[ { - \frac{{(X - X_i)_{{}}^{T} (X - X_i)}}{{2\sigma_{{}}^{2} }}} \right]}$$

Equation (3) is a weighted exponent summation of all pattern layer’s neurons, the neurons connection weights of the *i*th node in the pattern layer and the *j*th molecule in the summation layer is the *j*th unit in the *i*th output samples, the conversion formula is $S_{N_j} = \sum\nolimits_{i = 1}^{n} {y_{ij}P_i} \quad j = 1,2, \ldots ,k$.

Finally, the output neuron may provide the desired results, that is $\hat{y}_{i} = \frac{S_{N_j}}{S_D}\quad j = 1,2, \ldots ,k$.2.2. Niche Particle Swarm Optimization (NPSO).

PSO was first introduced in 1995 by the American social psychologist Kennedy and electrical engineers Eberhart. In the PSO algorithm, we think each individual as particles without mass and volume in D dimensional search space and flight with a certain speed (Li et al. 2009).

PSO algorithm’s speed and position evolution equations are:

4 $$\upsilon_{j} (t + 1) = \omega \upsilon_{j} (t) + c_{1} r_{1} (p_{j} (t) - \chi_{j} (t)) + c_{2} r_{2} (p_{g} (t) - \chi_{j} (t))$$

5 $$x_{j} (t + 1) = \nu_{j} (t + 1) + x_{j} (t)$$

where $\upsilon_{j} (t)$ express the particle $j$’s velocity in the $t$ th generation, $\omega$ = the inertia weight, $c_{1}$ = the cognitive factor, $r_{1}$ and $r_{2}$ are variables manually set to control convergence of swarm, $p_{j} (t)$ represents the individual history optimal location of particle *j*, $x_{j} (t)$ is the particle *j*’s location in the $t$ th generation, $c_{2}$ = social factor, $p_{g} (t)$ on behalf of the best position the swarm has obtained, $x_{j} (t + 1)$ indicate the particle *j*’s location in the $t + 1$-th generation.

In 2002, Brits etc. introduced niche technology into the PSO and developed Niche PSO (Brits et al. 2002). In order to keep the diversity of PSO, if multiple iterations of a particle in a continuous operation corresponding to adapt to changes in the value of a small amount, then this is the center of the particle, the particle radius construct with its nearest distance of a small round particles habitats. The radius of niche PSO is defined as

6 $$R_{{s_{j} }} = \hbox{max} \{ ||x_{{s_{j} ,g}} - x_{{s_{j} ,i}} ||\}$$

where $x_{{s_{j} ,g}}$ = the best particle in particle swarm $S_{j}$, $x_{{s_{j}^{{}} ,i}}$ indicate any one of the non-optimal particle in particle swarm $S_{j}$.

Algorithm could be progressed with two core operations:

(1) If the particles $x_{i}$ enter into the range of sub particle swarm $S_{j}$, expressed as $\left. {\left\| {x_{i} - x_{{s_{j} ,i}} } \right.} \right\| \le R_{{s_{j} }}$, then the particles will be assimilated by this NPS.

(2) If $S_{j}$ and $S_{k}$’s range are intersected, that is $\left. {\left\| {x_{i} - x_{{s_{j} ,i}} } \right.} \right\| \le \left| {\left. {R_{{s_{j} }} - R_{{s_{k} }} } \right|} \right.$, then the two sub-PS will be united into one.

### NPSO-GRNN steps for bid/no-bid decisions

Seven steps were employed to build the simulation model for bid/no-bid decisions, as follows:

*Step 1* population initialization and parameter initialization settings, the particle size is N, *c*_1_ is cognitive factor and *c*_2_ is social factor, iteration termination condition.

*Step 2* Since Bid/no-bid decision factor has a different meaning and a different physical dimension and magnitude, the original date needs for raw data normalized before GRNN neural network training. This paper introduced the ratio of compression method for processing the raw data, Formula can be expressed as:

7 $$T = T_{\rm min} + \frac{{T_{\rm max} - T_{\rm min} }}{{X_{\rm max} - X_{\rm min} }}(X - X_{\rm min} )$$

where X = raw data, $X_{\rm max}$ = the maximum of raw data, $X_{\rm min }$ = the minimum of raw data, T = the converted data, also known as target data, $T_{\rm max}$ = the maximum of target data, $T_{\rm min}$ = the minimum of target data.

Step 3 Determine the fitness function: using the output value and actual value’s variance of training samples F as the fitness function to find the best $\sigma$, the fitness function is:

8 $$F = \sqrt {\frac{1}{N}\sum\limits_{i = 1}^{N} {(\hat{y}_{i} - yi)^{2} } }$$

where $y_{i}$ = the actual value, $\hat{y}_{i}$ = the calculated value, the smaller value of F of training samples, the more conducive to iterative algorithm stops.

*Step* 4 Taking the study samples and particles into the GRNN neural network.

*Step* 5 Calculate the fitness value of each particle and retains optimal fitness and individual, check whether it comes the optimized conditions, if it reaches the error accuracy, then end. Otherwise, go to the next niche groups of particles to optimize, the current global extreme optimal of the particle populations’ optimal solution is the spread parameter of GRNN neural network.

*Step* 6 If the optimal value is not found, then form a new group space for the best individual niche groups of each particle retention, redefine individual niche populations, repeat steps (4).

*Step* 7 Optimized by the niche particle swarm optimization, when algorithm terminates, the position of the extreme points of the global is the smooth factor values of GRNN neural network for bid/no-bid decision model, then substitute it into GRNN neural network model to learn. In a word, it can be used for solving the prediction model.

## Data analysis and model implementation

### Variables

**Fig. 2:**
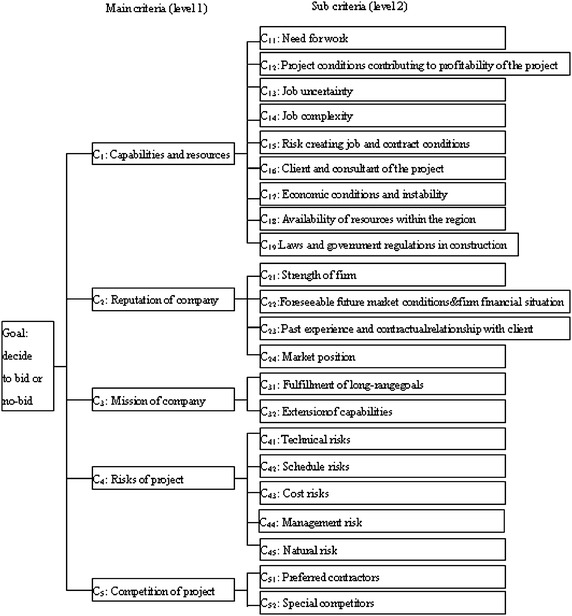
Variable definition

The aim of this part is to identify criteria affecting tendering decision. Owing to the high risk and cost of bidding for a large project, tendering documents are filtered and evaluated by a tendering board that have more than 30 years of experience in construction industry. Based on the aforementioned literature and interviewing in construction firms, the criteria list of 22 variables influencing bid making were collected. These variables were modified and grouped under the following five categories. The variables for project tender are shown as Fig. 2.

### Reduction

Rough set theory is a data analysis theory put forward by a polish mathematician Z.Pawlak in 1982. Let $X \subseteq U$ and $R$ as an equivalence relation. When $X$ repents for some basic categories ‘union and we said $X$ can be defined for $R$ (Definable R), otherwise, $X$ cannot be defined for $R$ (Indefinable R). Definable set $R$ can also be defined as accurate set (Exact Sets R), and indefinable R set can be called as Inexact Sets or Rough set. When there is an equivalence relation $R \in ind(K)$, and $X$ represents for $R$ accurate set, precise set $X \subseteq U$ is called of collection accurate set of K; As for any $R \in ind(K)$, $X$ is called rough set for $R$, $X$ is rough set of K.

**Fig. 3:**
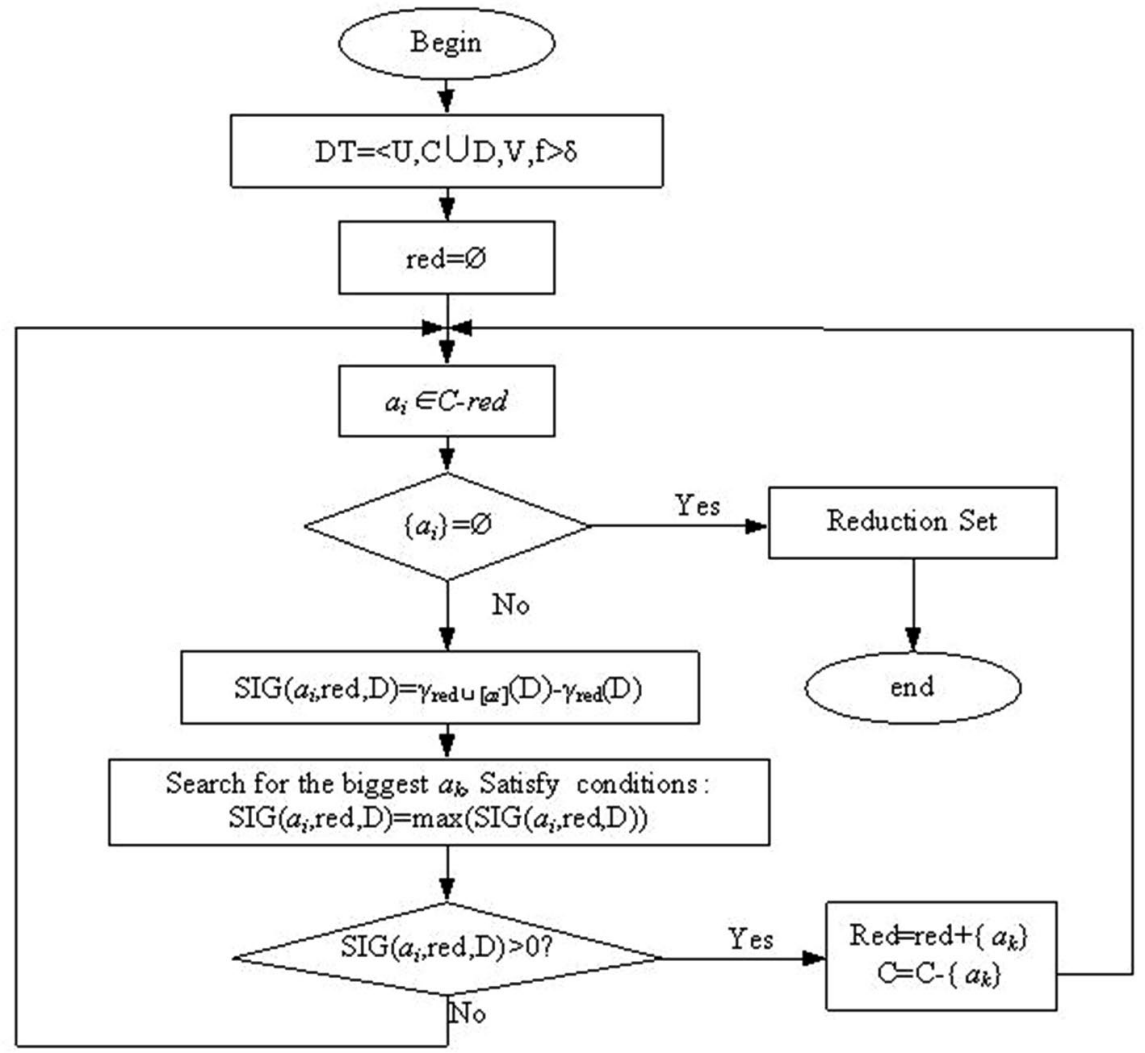
Flow chart of reduction

In general, the reduction of information system or decision table of knowledge is not the only one. In this paper, the optimal reduction is referred to a minimum number of attributes reduction. If there are multiple reduction with the minimum number of attributes at the same time, so the smallest attribute value combination reduction repress for the optimal reduction. The reduction under this rule is also known as the minimal reduction. The reduction technological process is summarized in Fig. 3.

### Variables processed by reduction

The attribute variables reduced by Rough Set (Pawlak 1994; Shi 2012) were shown in Table 1.

**Table 1:** Reduction results

Target	Variable
Bid/no-bid decision making ($U$)	Project demand degree ($U_{1}$)
	Project uncertainty ($U_{2}$)
	Strength of firm ($U_{3}$)
	Strategic target fulfillment ($U_{4}$)
	Technical risk ($U_{5}$)
	Cost risk ($U_{6}$)
	Preferred contractor ($U_{7}$)
	Special competitors ($U_{8}$)

### Simulating

Due to the impact of the variables considered are most difficultly quantitative descried, experts grading method was used. This method requires the respondents to grade the degree of importance of 8 variables that affect the tender decision making. Every variable is graded according to a 1-5 Likert scale, which “1” indicates not important, less and small etc., and “5” indicates most important, more and great etc.

Illustrating with example:

- Project demand degree: (less ~ more) corresponding (1~5)
- Project uncertainty: (small ~ great) corresponding (1~5)
- Strategic target fulfillment: (no meet ~ meet) corresponding (1~5)

## Results and discussion

This study proposed a novel approach of process bid/no-bid decision prediction while considering uncertainties and interdependencies among attribute and sub-attribute. Eight quantitative and qualitative factors having major impact on tending decision were identified by RS. Through investigation and analysis of the main features of tending cases in Beijing, strength of firm, project requirements, strategic target fulfillment, project uncertainty, cost risk, technical risk, preferred contractor and special competitors were as inputs of GRNN neural network, tender decision was as output. We take the above eight variables impacting bid/no-bid decision as input of NPSO-GRNN neural network, tendering demand as output, And take 20 cases of 2015 as the network data of training sample and the network data of prediction test samples. A computer program has been developed for training and predicting process utilizing MATLAB. Setting the size of particles in particle swarm niche N = 30, cognitive factors $c_{1}$ = 2 and social factors $c_{2}$ = 2. Iteration termination condition is training error of 10-4 or the maximum number of iterations 100.

Using trained of NPSO-GRNN neural network model and three other ANN models for tender decision in Beijing to predict. Taking the absolute value of the average relative error of MAPE (Mean Absolute Percentage Error) as a measure of prediction accuracy indicators, the formula for MAPE is

9 $$MAPE = \frac{1}{n}\sum\limits_{i = 1}^{n} {\left| {\frac{{y_{i} - \hat{y}_{i} }}{{y_{i} }}} \right|}$$

In formula (9), $y_{i}$ indict actual value, $\hat{y}_{i}$ indict calculated value.

In order to verify the superiority of proposed approach for bid/no-bid decision-making, the simulation example (as shown in Table 2) was used to train and test different types of neural network models, including RS- NPSO-GRNN, NPSO-GRNN, RS- GRNN (integrated algorithm of RS and GRNN neural network), GRNN and BPNN (back-propagation neural network), the relative error of prediction and training MSE results shown in Table 3. As shown in Table 3, both NPSO-GRNN neural network model and BP neural network model of MAPE were controlled at less than 5 % in training and prediction, the results achieve very high accuracy. The established of RS-NPSO-GRNN neural network model of urban domestic water prediction model is feasible, high forecast accuracy and more stability. Comparing with the BP neural network model, RS-NPSO-GRNN neural network model also has a fast convergence, few of adjust parameters and easy to local minima, etc.

**Table 2:** Sample distribution by tender need

Bid.	$U_{1}$	$U_{2}$	$U_{3}$	$U_{4}$	$U_{5}$	$U_{6}$	$U_{7}$	$U_{8}$	Tender decision
1	0.8000	0.4400	0.2000	0.2000	0.2000	0.5667	0.400	0.7559	Stronger
2	0.8000	0.4400	0.2000	0.2000	0.2000	0.5667	0.4400	0.7559	Strongest
3	0.2000	0.2000	0.8000	0.8000	0.2000	0.4333	0.5600	0.3500	Stronger
4	0.8000	0.4400	0.2000	0.8000	0.8000	0.5000	0.4400	0.7559	Weaker
5	0.3912	0.5360	0.8000	0.5000	0.8000	0.5667	0.4400	0.8000	Moderate
6	0.8000	0.4400	0.2000	0.6500	0.8000	0.6000	0.3200	0.7559	Weaker
7	0.3912	0.5360	0.8000	0.3500	0.2000	0.4667	0.4400	0.8000	Strongest
8	0.2000	0.2000	0.8000	0.8000	0.2000	0.2667	0.6800	0.5000	Strongest
9	0.8000	0.4400	0.2000	0.8000	0.6000	0.7667	0.2000	0.7559	Weaker
10	0.8000	0.4400	0.2000	0.2000	0.4000	0.5333	0.4400	0.7559	Weaker
11	0.2743	0.2720	0.2000	0.3500	0.2000	0.6333	0.3200	0.7559	Weaker
12	0.3912	0.5360	0.8000	0.3500	0.2000	0.4667	0.4400	0.8000	Stronger
13	0.2000	0.2000	0.8000	0.8000	0.2000	0.2667	0.6800	0.5000	Stronger
14	0.3912	0.5360	0.8000	0.5000	0.8000	0.5667	0.4400	0.8000	Moderate
…	…	…	…	…	…	…	…	…	…
38	0.4124	0.4400	0.5000	0.5000	0.2000	0.6667	0.3200	0.7559	Weakest

**Table 3:** Comparison results of identification performance based on different methods

Indexes	GRNN	BP	RS-GRNN	NPSO-GRNN	RS-NPSO-GRNN
Training accuracy (%)	88.46	89.33	96.33	96.67	98.33
Training error (%)	2.71	2.76	1.76	1.82	1.56
Training MSE	0.1035	0.1104	0.0421	0.04017	0.0112
Testing accuracy (%)	86.00	86.01	92.00	92.15	94.00
Testing error (%)	4.53	4.49	2.51	2.46	2.12
Testing MSE	0.2013	0.1895	0.08014	0.07127	0.01879
Simulation time (s)	28.27	28.36	21.76	25.15	21.07

## Conclusions

Aiming at aiding bid/no-bid decision making, this paper introduced a novel identification model through integration of rough sets(RS) and GRNN, with NPSO algorithm to optimize the smooth factor GRNN neural network and improve the prediction accuracy and convergence of networks. This method comprehensively considers various parameters that affect the tender decision. Rough sets(RS) were used to reduce the factors. MIBARK algorithm is applied in attribution reduction to simplify the network input dimension number. Furthermore, the NPSO algorithm is proposed to realize the optimization of GRNN parameters. A simulation example is provided and some comparisons with other ANN algorithms are carried out. The results show that the model proposed in this paper exhibits fairly good prediction accuracy in the same test sample, that is, the value of MSE is only 0.0112.The results of examples show that using NPSO-GRNN neural network prediction model for Bid/no-bid decision prediction is reasonable and feasible. NPSO-GRNN neural network model offers a novel model and method to predict Bid/no-bid decision.
